# Tensed Emotions, Evolution, and Time

**DOI:** 10.1007/s11406-017-9927-8

**Published:** 2017-11-22

**Authors:** Olley Pearson

**Affiliations:** Department of Philosophy, Durham University, 50 Old Elvet, Durham, DH1 3HN UK

**Keywords:** Time, Tense, Emotions, Evolution, Prior

## Abstract

Prior showed that one could be relieved that the exams were over and not that they finished before a certain date or before a certain entity. One might think that these differences in relief are responsive to differences in the world so that there is more to the exams being over than them finishing before a certain date or entity: there is a metaphysical tense. However, some have argued that these issues do not have any implications for the metaphysics of time because they can be explained with an evolutionary account: emotions can have a causal influence on events that come after them but not on events that come before them and so it evolutionarily advantageous that emotions that concern events after them feel and evaluate differently to those that concern events before them. Here I will argue that to date these evolutionary accounts remain insufficient and emotions continue to put pressure on those who deny there is a metaphysical tense. Prior’s case shows that tensed and tenseless emotions differ in appropriateness. This asymmetry of value is not explained by the evolutionary account because whether or not an emotion is tensed is quite independent of whether it comes before or after something.

In 1959 Prior presented his thank goodness argument showing that one could be relieved that the exams were over and not that they finished before a certain date or before a certain entity such as the relief itself (Prior 1959). This raises a number of issues to do with the meaning and appropriateness of emotions. One might think that these differences in relief are responsive to differences in the world. There is more to the exams being over than them finishing before a certain date or entity: there is a metaphysical tense. However, some have argued that these issues do not have any implications for the philosophy of time because they can be explained with an evolutionary account. Emotions can have a causal influence on events that come after them but not on those before them. Therefore, it makes sense that emotions that concern events after them feel and evaluate differently to those that concern events before them.

Here I will argue that to date these evolutionary accounts remain insufficient and that Prior’s argument has a lingering import for our metaphysics as well as for our semantics. In Section 1 I outline the linguistic and potential metaphysical import of our emotions. In Section 2 I describe a current tenseless evolutionary account of these matters. In Section 3 I argue that the evolutionary account centres on a difference between an emotion coming after something and an emotion coming before that thing. Thus it might explain why two emotions differing in this way differ in appropriateness. Finally, in Section 4 I argue that Prior’s case shows that tensed and tenseless emotions differ in appropriateness. This asymmetry of value is not accounted for by the current evolutionary story because whether or not an emotion is tensed varies independently of whether it comes before or after something. The conclusion of the paper will be that those who want to deny that there is a metaphysical tense cannot rely on the current evolutionary account to address issues raised by emotions and Prior’s argument, however, it remains an open possibility whether a better evolutionary account can be given.

## A Difference in Meaning

The case at hand is one in which Prior says “Thank goodness that’s over” in reference to the ending of a period of examinations (I will often refer to these explicitly to avoid complications surrounding demonstratives). When he says this Prior is unaware that the time is 4 pm on the 15th of June 1954 and so he would not happily say the tenseless “Thank goodness the exams end before 4pm on the 15^th^ of June 1954”. Prior is also not overly concerned that he has this relief or makes this utterance after the end of the exams and he would not say the tenseless “Thanks goodness the exams end before this utterance [relief/belief]” or “Thank goodness the exams end before NN”, where ‘NN’ is a name or description of the utterance [relief/belief].

An initial point of import of these facts is that they show the first utterance cannot be translated by any of the others. They must mean different things as they cannot be used in the same circumstances to express the same emotions. This is important as at the time of Prior’s giving this argument some believed that tensed utterances could be translated by tenseless ones. The tenseless translations which were offered either inserted the date of the utterance/relief/belief or a specific reference to the utterance/relief/belief. For example, an utterance of ‘MM is past’ made at t, will be translated by ‘MM is before t’ or ‘MM is before this utterance’ or ‘MM is before NN’, where ‘NN’ refers to the utterance itself.

Many of those who believed that tensed utterances could be translated in this way adopted a *tenseless theory of time* according to which tensed and tenseless beliefs and utterances concern the same facts —tenseless ones.^1^ This view is in opposition to a *tensed theory of time* according to which tensed beliefs and utterances concern something about the world —a metaphysical tense— not captured by tenseless ones. If translation is possible the tensed view is mistaken. Thus, Prior’s argument had the further import of blocking one defence of the tenseless theory. However, more recently it is widely recognized that tensed and tenseless utterances and beliefs might capture the same facts despite having different meanings.^2^ The surface semantic difference does not vindicate the tensed view.

## A Difference in Appropriateness and an Evolutionary Account Thereof

Prior appears to be appropriately relieved that the exams are over and this relief appears to be necessarily tensed. That is, it must be expressed with tensed language. It is relief that the exams are *over* not that they end before a certain date or entity. I use ‘tensed emotion’ to refer to an emotion like Prior’s which is naturally expressed with tensed language. I use ‘tenseless emotion’ to refer to an emotion naturally expressed with tenseless language, such as the emotions Prior denies he has. Tensed emotions can thus be distinguished from tenseless emotions through their expression precisely as Prior’s case makes clear. Further, recognizing this does not commit one to a particular metaphysics of time (as the case of belief alluded to just above makes clear). A difference in expression or meaning needn’t coincide with a difference in content or what is represented. Tensed emotions are thus something that one can and should accept whether or not one adopts a tensed view of time.

Because the tensed utterances considered by Prior are taken to express emotions his argument raises issues to do with the appropriateness of such utterances and emotions. Further, the appropriateness of Prior’s emotion is bound up with time. It is appropriate to be relieved that a bad experience is past but not that one is future.^3^ Relief is temporally orientated and this is a characteristic shared by many other emotions. For example, it is appropriate to grieve a past death but not a future one, appropriate to fear a future danger but not a past one, and so on. I shall refer to characteristics like these as *asymmetries in appropriateness*.^4^

This has led some to see further significance in Prior’s case. If the tenseless view of time is correct a future discomfort [or death etc.] is in itself no different from a past one. Therefore, we appear to have no ready account for asymmetries in appropriateness. For example, no ready account for why it is appropriate to be relieved about a past discomfort and not about a future one. Prima facie the tensed view does not suffer this difficulty. It allows a difference between a past, a present, and a future discomfort [or death etc.] which might be taken to ground the asymmetry in appropriateness. In this way Prior’s case puts the tensed view at an advantage over the tenseless view.^5^

Recently a number of writers have responded that one can readily account for these asymmetries of appropriateness with an evolutionary story which is plausibly compatible with a tenseless view. I will raise doubts about this response below, but first it is important to make clear what it involves.

Maclaurin and Dyke (2002; Dyke and MacLaurin 2013) have tried to give an evolutionary account of the asymmetries of appropriateness. Their account is based on causal asymmetry: effects must follow their causes in time. They consider the case of fear, which they take to be a state that readies the body for action. Fear can only help one avoid danger that comes after that fear. Thus fear only has good consequences for one’s safety if it concerns future dangers and it is in considering future dangers that fear will be evolutionarily advantageous. Therefore, if we take our evaluations of the appropriateness of emotions of fear to be based on their evolutionary advantage, it makes sense that we evaluate fear that concerns future danger to be appropriate and fear that concerns past danger to be inappropriate. Being afraid of a danger I was in yesterday but am no longer in will not help me avoid that danger and so is not appropriate.

Maclaurin and Dyke suggest that something like this pattern can also account for our evaluation of other emotions. This will not always be straightforward. For example, when we consider emotions such as relief that are appropriate when they concern the past and not when they concern the future. But, it will often be possible to tell some sort of related story. For example, referring to an emotion’s causal role in preventing us repeating mistakes.^6^ One can account for asymmetries of appropriateness whilst adopting a tenseless theory of time because one can ground this asymmetry on a causal asymmetry which is relevant to evolution. This causal asymmetry does not rely on there being a metaphysical tense. It, merely relies on their being a difference between an emotion coming before an entity and that emotion coming after that entity.

Suhler and Callender (2012) go slightly further than Maclaurin and Dyke in showing how their account fits other hypotheses concerning human preferences. Others, such as Oaklander (1993), offer an evolutionary account in line with Maclaurin and Dyke’s though with less detail. But at root they all agree that if the asymmetries of appropriateness can be accounted for, this can be done with reference to a causal asymmetry and evolution. (The targets of my discussion are the various tenseless accounts that share this last assumption but do not add much to it.)

## The Evolutionary Account Neglects the Crucial Tensed/Tenseless Asymmetry

The evolutionary account is based on a causal asymmetry which is an asymmetry between something preceding and something following an emotion. Emotions can only causally affect things that follow them. This means that what the evolutionary account might explain is a difference between an emotion that concerns something that follows it and an emotion that concerns something that precedes it. However, as I will now argue, this leaves many of the issues raised by emotions and Prior’s case unexplained.

We must distinguish four cases because there are two ways that an emotion might concern something that follows it and two ways that an emotion might concern something that precedes it. The emotion might be tensed or tenseless. For example:

- In Case 1, Prior has a tenseless emotion of relief that concerns a discomfort that follows it. He might express such an emotion by saying “Thank goodness the examinations end at 5pm on the 15^th^ of June 1954”.
- In Case 2, Prior has a tensed emotion of relief that concerns a discomfort that follows it. He might express such an emotion by saying “Thank goodness the examinations will continue”.
- In Case 3, Prior has a tenseless emotion of relief that concerns a discomfort that precedes it. He might express such an emotion by saying “Thank goodness the examinations end at 4pm on the 15^th^ of June 1954”.
- In Case 4, Prior has a tensed emotion of relief that concerns a discomfort that precedes it. This is the sort of case Prior actually suggests to occur and he might express that emotion by saying “Thank goodness that’s over”.

The causal asymmetry is an asymmetry between something preceding and something following an emotion. The causal asymmetry is therefore blind to any difference between Case 1 and Case 2 as they are both cases of a relief that concerns a discomfort that follows it. Similarly, the causal asymmetry is blind to any difference between Cases 3 and 4. Because the evolutionary account of our evaluation of emotions is based merely on this causal asymmetry it must therefore evaluate Cases 1 and 2 equally, and both differently from Cases 3 and 4 which are likewise evaluated equally. (What the relief concerns is also of import to its evaluation but this can be ignored here as it can be assumed that in Cases 1 and 2 the relief concerns the same thing despite the related utterances differing in meaning and similarly for Cases 3 and 4. My talk of what an emotion concerns is talk of the facts an emotion concerns or the states of the world the emotion is a response to. If the tenseless theory is correct tensed and tenseless emotions concern the same thing whether or not one takes them to share propositional content. One might try to account for a difference in the appropriateness of tensed and tenseless emotions through a difference in their propositional content or what they concern, however, my point is that this is not the simple causal/evolutionary account which is offered.)

In short, if the evolutionary account explains anything, it is that Cases 1 and 2 are cases of inappropriate emotions and Cases 3 and 4 are cases of appropriate emotions (or perhaps vice versa). However, as I will make clear in the next section, Case 4 is the only example of an appropriate emotion and therefore the evolutionary account is not an adequate account of our evaluation of emotions. Reference to a causal asymmetry alone cannot provide a full account of emotional appropriateness.

## Only Past-Tense Relief Is Appropriate

Relief is appropriate towards a past discomfort but not a future one (thus Case 4 is appropriate and 2 is inappropriate). It is appropriate to be relieved that a discomfort *has ended* but not that it *will continue/end*.^7^ This means that the appropriateness of the relief is bound with whether or not a discomfort is past and implies that it is in being sensitive to this that relief is able to be appropriate. The causal/evolutionary account is able to account for this need for appropriate relief to concern the past discomforts as opposed to future ones. All parties agree relief ought to concern the past. They differ over what being past amounts to.

Crucially, however, if appropriate relief must be sensitive to the past, then not only is relief that concerns the future inappropriate [Case 2] but so too is tenseless relief [Cases 1 and 3]. The reason for this is simple. Tenseless relief needn’t be sensitive to whether or not the discomfort it concerns is past. In fact, it is precisely relief that is not sensitive to whether the discomfort it concerns is past just as tenseless beliefs are precisely those that are not sensitive to whether their subjects are past/present/future. More specifically, one can have a tenseless belief about a discomfort without knowing whether or not it is past and one can have a tenseless relief without knowing if the discomfort it concerns is past. But, if appropriate relief must concern the past one is not forming an appropriate relief if one does not know whether what it concerns is past. Prior’s relief is inappropriate if it concerns the future and it is equally inappropriate if for all he knows it concerns the future.

There is a slight slide here from ‘the emotion is not sensitive to tense’ to ‘the agent doesn’t know the tense’. However, this transition is allowed for by the fact that what makes it appropriate to express an emotion with tensed language or tenseless language is the state of the mind of the emotional agent. Prior might not know the tenseless time of his discomfort’s ending but whether he does or not his relief concerns the discomfort being past. If an agent knows whether a discomfort is past and is responding to that that is reflected in the expression of the emotion. It is what I have been calling a tensed emotion. A tenseless emotion is precisely one where the pastness of a discomfort is not playing a role either because one doesn’t know whether the discomfort is past or because one doesn’t care. Either way, a tenseless emotion isn’t sensitive to whether the discomfort it concerns is past as a case of appropriate relief must be.

It is important to note that the comments of the preceding paragraphs do not presuppose a tensed theory of time. Rather, all parties must agree some things are past and others are not, and that one can be unaware whether or not something is past. A tenseless theory that denied this would be too far from everyday experience to be a strong candidate for truth which is why modern tenseless theories are careful to allow many tensed statements to be true.

At this point one might respond that so long as an emotion concerns a discomfort that precedes it it will be appropriate, whether or not one knows whether the discomfort is past. But, this is incorrect because if one doesn’t know whether the discomfort is past one’s relief will not be sensitive to whether the discomfort precedes it in the appropriate manner.

Consider the following case. Let us suppose that emotions along with other mental states are brain states that can be measured by brain scans and can be represented on a screen.^8^ Further, let us suppose that Prior is taking part in an experiment which involves a number of individuals with micro scanners implanted in their sculls which transmit to a lab where all of these individuals’ brain states are measured and are often displayed. One day Prior might be in the lab looking at a screen which displayed a number of his brain states. These might all be states of discomfort (suppose he has regular headaches). He might look at one of these states, D1, and recognizing it as a discomfort of his feel relief. D1 is a headache Prior had the previous day so his relief concerns a discomfort that precedes it and is appropriate according to the response I am here considering. However, Prior’s relief is not appropriate on this occasion because for all he knows he is looking at a scan of his current headache and discomfort, so something he should not feel relief towards. Here is a case of inappropriate relief concerning a discomfort that precedes it.

We can elaborate on the example to show that Prior’s relief is still inappropriate even if he knows that it is preceded by the discomfort. Suppose that the lab technician Sue walks in and chats to Prior. She tells him that she will put an image displaying an emotion, R1, on the screen for him to see soon. Further, she tells him that D1 precedes R1, though she tells him nothing more about R1. Prior continues looking at D1 and feeling relief. As it happens though, R1 is Prior’s current state of relief. Here, Prior has relief that concerns a discomfort that precedes it and that he knows precedes it, yet Prior’s relief is still inappropriate because for all he knows that discomfort is one he is feeling presently.

If we instead suppose that Prior takes the discomfort to be past, perhaps the particularly nasty headache he had yesterday, then his relief appears to be appropriate. Thus, we see that relief must be sensitive to whether a discomfort is past, not merely to a discomfort that precedes it or that is taken to precede it. Relief must be tensed to be appropriate.

This example highlights that the appropriateness of the emotion is not merely causally fixed. A relief is not appropriate simply because it is caused by a discomfort that precedes it. Though this shouldn’t be a surprise given some emotions are future orientated and so cannot be caused by what they are appropriately responsive too. My anticipation about my supper is appropriate because that supper is future but not because that supper causes my anticipation. This indicates that one’s awareness of one’s environment plays a role in the appropriateness of one’s emotions. However, there is no need to go into detail about this to show the causal/evolutionary account to be inadequate. Quite why some emotions —such as Prior’s relief— are best expressed with tensed language does not need to be shown to establish that only a subset of tensed reliefs can be appropriate.

Thus Case 4 is a case of appropriate emotion and Cases 1, 2, and 3 are not. The distinction between appropriate and inappropriate relief does not simply follow the distinction between relief that concerns a discomfort that precedes it and relief that concerns a discomfort that follows it. The causal asymmetry referred to by the evolutionary account to explain the appropriateness of relief is therefore not sufficient to account for this asymmetry of appropriateness. Analogous points arise for other emotions with a temporal orientation and asymmetry of appropriateness. If one does not take a danger to be present/future then one’s fear is not appropriate. If one does not take a pleasure to be future then one’s anticipation is inappropriate. And so on.

The comments just made are not enough to establish the tensed theory. However, they do show that if Prior’s argument fails to justify the tensed theory, this is not because of the current causal evolutionary accounts of emotions found in discussions of the metaphysics of time.

Admittedly, many of those who offer causal evolutionary accounts focus on an asymmetry of value between an emotion that concerns something that precedes it and one that concerns something that follows it. They do not focus on an asymmetry of value between tensed and tenseless emotions. However, as I have shown, these two asymmetries are linked.^9^ Further, Prior actually presents his case by distinguishing tensed and tenseless emotions which all concern a discomfort that precedes the emotion. That is, Prior’s case explicitly concerns the tensed/tenseless asymmetry. Therefore, it is no defence of, for example Maclaurin and Dyke, to say that they were not trying to address the issues I raised. On the contrary, MacLaurin and Dyke (2002, 292) claim that the evolutionary account that they provide is very plausible and removes the onus on the tenseless theory that was placed on it by Prior. My argument shows that this is false. If Prior has placed an onus on the tenseless theory, then this is not removed by the causal evolutionary story they have told to date.

## Conclusion

Prior’s thank goodness argument made a strong point against those that believed tensed utterances/beliefs could be translated by tenseless ones. Prior’s argument also put prima facie pressure on those who adopt a tenseless theory because they must account for why tensed and tenseless emotions differ in appropriateness if they concern the same [tenseless] facts. This pressure has not been relieved by the causal evolutionary accounts offered to date. These accounts fail to confront the asymmetry between tensed and tenseless emotions instead focusing on an apparent asymmetry between emotions that concern things which precede them and emotions that concern things which follow them. If there is pressure put on the tenseless theory by the fact that a mere difference in the tense of the expression of an emotion alters whether the emotion is appropriate, then this pressure has not been alleviated by the causal evolutionary accounts offered. This is not to deny any evolutionary account can be given, but to show that we must set about giving a more adequate one. Consequently, the causal evolutionary account has not yet shown Prior’s case to be over after all.

## Footnotes

1 Cf. for example, Goodman 1951.

2 Cf. Oaklander and Smith 1994.

3 Mellor 1981 challenges this idea with the existence of masochists that are supposedly relieved when a pain starts. I, like MacBeath 1983, am not happy with this characterisation. Pain for these people is not a discomfort. However, if need be we could consider the case of grief instead, where there are no such exceptions. Hoerl 2013 distinguishes two forms of relief, temporal and counterfactual. If this distinction were accepted, my argument only applies to the former sort but this is the sort at issue.

4 These asymmetries are widely recognised in the literature. Cf., for example, Oaklander 1993 and Maclaurin and Dyke 2002.

5 For a theory of the normativity of emotions that might fit these ideas, cf. Deonna and Teroni 2012: 96–98.

6 Though, Maclaurin and Dyke admit that they still struggle with some cases, such as grief.

7 Even if knowing a discomfort will end may alleviate one’s anxiety, being relieved to have learnt it will end is not the same as being relieved it will end.

8 My argument does not require the supposition that mental states are brain states, merely that one can know them third-personally and without knowing their tense. This is something that can and ought to be accepted by most forms of physicalism and dualism.

9 Moreover, when dealing with tenseless emotions of relief, their appropriateness does not differ whether or not they precede or follow a discomfort.
